# Complex posttraumatic stress disorder in adolescence: A two-year follow-up study

**DOI:** 10.1177/13591045231187975

**Published:** 2023-07-04

**Authors:** Evaldas Kazlauskas, Agniete Kairyte, Paulina Zelviene

**Affiliations:** Center for Psychotraumatology, Institute of Psychology, 54694 Vilnius University, Vilnius, Lithuania

**Keywords:** Complex PTSD, adolescence, ICD-11, trauma, longitudinal

## Abstract

**Background:**

Complex posttraumatic stress disorder is a new diagnosis in the 11th edition of the International Classification of Diseases (ICD-11). There is a need for a better understanding of complex PTSD in children and adolescents.

**Objective:**

The study aimed to estimate the factors associated with chronic complex PTSD versus recovery of complex PTSD in adolescents in a 2-year follow-up study.

**Method:**

In total, 66 adolescents, mean age 14.5, 73% female, identified as having complex PTSD using self-report at baseline recruited from a general population sample, were included in the study. The International Trauma Questionnaire - Child and Adolescent Version (ITQ-CA) was used for the assessment of complex PTSD.

**Results:**

Overall, 36% of the study sample has been identified as having chronic complex PTSD over 2 years, 10% met the criteria for PTSD at a 2-year follow-up, and 54% recovered. A higher risk for chronic complex PTSD was associated with exposure to more traumatic events and more life-stressors over the 2 years, low social network, low positive social support, bullying at school, and loneliness.

**Conclusion:**

The study found that around one-third of the traumatized youth had a prolonged trajectory of complex PTSD symptoms, which were associated with negative life experiences and social difficulties.

## Introduction

The newest edition of the International Classification of Diseases (ICD-11), in addition to posttraumatic stress disorder (PTSD), included a new diagnosis of complex PTSD (World Health Organization, 2018). Complex PTSD may develop following traumatic event or events, most often prolonged or repetitive, such as sexual abuse, torture, and prolonged domestic violence. Complex PTSD symptom structure includes three PTSD core elements (1) re-experiencing, (2) avoidance, and (3) persistent perception of threat, and three complex PTSD-specific symptoms of (4) severe problems in affect regulation, (5) persistent negative beliefs about oneself, and (6) persistent difficulties in relationships (World Health Organization, 2018).

The differentiation between PTSD and complex PTSD has implications for research and clinical practice. Clinical practice should be able to identify and understand complex PTSD manifestation and risk factors, and clinicians are expected to apply therapies specific to complex PTSD. By separating these two trauma-related disorders, the prevalence and risk factors for PTSD and complex PTSD differ, and assessment tools for complex PTSD are needed. Furthermore, ICD-11 describes that complex PTSD can be diagnosed across the lifespan, including children and adolescents, using the same criteria (World Health Organization, 2018). Moreover, ICD-11 indicates that children supposedly can be at a higher risk for complex PTSD than adults (World Health Organization, 2018). Complex PTSD in children and adolescents should be diagnosed taking into consideration the developmental stage in the assessment of symptom expression (Maercker et al., 2022). However, current knowledge about complex PTSD has mostly been accumulated from adult studies (Brewin et al., 2017; Redican et al., 2021).

The number of studies on complex PTSD in children and adolescents has been growing in the last several years, but these studies are still scarce (Cloitre et al., 2021). Studies reported evidence for the validity of ICD-11 PTSD and complex PTSD factor structure in adolescence (Haselgruber et al., 2020; Kazlauskas et al., 2020; Li et al., 2021; Sachser et al., 2021; Sachser, Keller, et al., 2017; Tian et al., 2021). Studies also provided information on psychometric properties of complex PTSD assessment tools in children and adolescents (e.g., Redican et al., 2022; Rossi et al., 2022; Sachser et al., 2022), and reported prevalence and risk factors for complex PTSD (e.g., Daniunaite et al., 2021; Elliott et al., 2020; Haselgruber et al., 2020; Redican et al., 2022).

The majority of complex PTSD studies have been conducted using self-report measures, such as the International Trauma Questionnaire – Child and Adolescent version (ITQ-CA) (Kazlauskas et al., 2020). The Child and Adolescent Trauma Screen 2 (CATS-2), recently updated to measure ICD-11 complex PTSD in addition to DSM-5 PTSD (Sachser et al., 2022), is also available. Prevalence of probable complex PTSD and PTSD in non-clinical samples based on self-report was found to be 3.4% and 1.5% in 11-19-year-olds (*N* = 507) in Ireland (Redican et al., 2022), 4.1% and 9.3% in high school students aged 18+ (*N* = 1010) in Italy (Rossi et al., 2022), 12.3% and 5.2% in 13-18-year-olds (*N* = 832) in Lithuania, and 4.1% and 2.3% in 12-18-year-olds (*N* = 914) in Japan (Kazlauskas et al., 2022). Multiple trauma exposure, especially sexual trauma (Redican et al., 2022), school and family problems (Daniunaite et al., 2021), and female gender (Kazlauskas et al., 2022; Redican et al., 2022) differentiated between complex PTSD and PTSD.

The majority of complex PTSD research is cross-sectional. One study found 2.4% and 7.2% prevalence of ICD-11 complex PTSD and PTSD symptoms in youth aged 8–17 after a single-event trauma in the 9^th^ week after single-event trauma (Elliott et al., 2020). There is a lack of knowledge on trajectories of ICD-11 complex PTSD and PTSD in children and adolescents, and factors that contribute to recovery of complex PTSD over time. The present longitudinal study aimed to estimate the factors associated with chronic complex PTSD versus recovery of complex PTSD in adolescents in a 2-year follow-up study. Based on the previous studies, we explored the role of trauma exposure and social factors on complex PTSD. The current study was built up on the previous study in Lithuania, which reported risk factors for complex PTSD in a Lithuanian general population-driven sample.

## Methods

### A study design, procedures, and dataset

Data for the study was extracted from a dataset of the longitudinal study “Stress and Resilience in Adolescence” (STAR-A). The STAR-A study aims to evaluate psychosocial functioning and posttraumatic effects of childhood abuse and traumatic experiences in a Lithuanian general population adolescent sample. The study was approved by the Research Ethics Committee for Psychological Research at Vilnius University. The quota sampling method was used to recruit adolescents aged 12–16 in Lithuania at baseline (T1). At T1 all students in grades 6 to 9 in selected schools across various regions of Lithuania were invited to participate in the study. The parental written informed consent (response rate 56.8%), and adolescent’s ascent were obtained before data collection. The baseline sample included a cohort of 1299 adolescents aged 12–16 (*M* = 14.25, *SD* = 1.27) with data collected in March-June 2019 (T1), and T2 (*N* = 832) in March-June 2021 participated in the study. More detailed methods and procedures of the STAR-A have been published previously (Daniunaite et al., 2021; Kazlauskas et al., 2020, 2022; Zelviene et al., 2020). The target sample for this complex PTSD study was 108 adolescents who met the diagnostic criteria for complex PTSD at T1 (Daniunaite et al., 2021), 66 of whom were reached at T2, resulting in a 38.9% drop-out rate.

### Participants

In total, 66 adolescents (70% female) who participated in T1 and T2 and had complex PTSD at T1 were included in this study. At the T1, the mean age of the sample was 14.48 (*SD* = 1.17), most of the participants did not experience financial difficulties in the family (86.4%), 90.9% of participants' mothers and 83.3% of fathers were employed, 37.9% of participants both parents had a university degree. At the T2 measurement, most participants (77.3%) answered that they did not receive any psychological services in the past year. For detailed sociodemographic characteristics of the sample, see Table 1.

**Table 1.** Sociodemographic information of the study sample (*N* = 66).

Variable	Total^a^ *N* = 66		Chronic complex PTSD *n =* 24		Recovery *n =* 36		χ^2^ (*df*)^b^
	*n*	%	*n*	%	*n*	%	
Gender							
Male	18	27.3	5	20.8	13	36.1	1.61 (1)
Female	48	72.7	19	79.2	23	63.9	
Age							
*M* (*SD*)	14.48 (1.17)		14.58 (1.14)		14.42 (1.25)		*t* (58) = −0.54
Range	12–16		12–16		12–16		
Financial difficulties in family							
No	57	86.4	20	83.3	31	86.1	0.09 (1)
Yes	9	13.6	4	16.7	5	13.9	
Mother employed							
Yes	60	90.9	4	16.7	2	5.6	1.98 (1)
No	6	9.1	20	83.3	34	94.4	
Father employed							
Yes	55	83.3	19	79.2	30	83.3	0.98 (2)
No	6	9.1	2	8.3	4	11.1	
Do not know	5	7.6	3	12.5	2	5.6	
Parent university degree							
No	8	12.1	5	20.8	3	8.3	4.43 (3)
One	19	28.8	8	33.3	9	25.0	
Both	25	37.9	6	25.0	18	50.0	
Do not know	14	21.2	5	20.8	6	16.7	
Professional support services (T1)							
No	50	75.8	17	70.8	28	77.8	0.37 (1)
Yes	16	24.2	7	29.2	8	22.2	
Psychological therapy (T2)							
No	51	77.3	13	54.2	32	88.9	9.27 (2) **
One or several sessions	4	6.1	3	12.5	1	2.8	
Several or more months	11	16.7	8	33.3	3	8.3	

**p ≤ .01

^a^includes six PTSD cases at T2.

^b^recovery versus chronic complex PTSD group comparison statistics.

### Measures

#### Trauma exposure

We used the Child and Adolescent Trauma Screen (CATS, Sachser et al., 2017) for measuring exposure to potential lifetime traumatic events (PTEs). The CATS is a list of 14 various PTEs. Adolescents were asked to indicate if they experienced these events, with probable dichotomous answers of *yes*/*no*. At the 2-year follow-up, the CATS list was used to assess exposure to PTEs over the last 24 months.

#### Posttraumatic stress reactions

The International Trauma Questionnaire – Child and Adolescent version (ITQ-CA, Kazlauskas et al., 2020) was used to measure complex PTSD symptoms and probable complex PTSD. The ITQ-CA resembles adult ITQ with the wording of items adjusted to children aged 7–17 years. The ITQ-CA comprises 12 symptom items: (1) six PTSD symptom items measuring three symptom clusters of re-experiencing, avoidance, and sense of threat with two items each, and (2) six complex PTSD-specific disturbances in self-regulation (DSO) items clustered into three symptoms of a negative self-concept, difficulties in relationships and emotion dysregulation. The ITQ-CA items assess how much each symptom bothered a person during the past month on a five-point Likert scale, ranging from *Never* (= 0) to *Almost all the time* (= 4). After each PTSD and DSO symptom evaluation, participants were asked if these symptoms interfered with friends or family relationships, school, or other important areas of life and general happiness.

In this study, we used the total PTSD score, DSO score, and complex PTSD score, which is the combined total of the PTSD and DSO scores. The total score of PTSD and DSO may range from 0 to 24 each, and the total score of complex PTSD may range from 0 to 48. Probable PTSD and complex PTSD was identified using the ITQ-CA diagnostic algorithm used in previous studies (Kazlauskas et al., 2022). The participants were screened positive for PTSD if at least one item in all PTSD symptom clusters were ≥ 2, and at least one area of life was interfered with PTSD symptoms. Probable complex PTSD was considered if participant met diagnostic criteria for PTSD, and at least one item in all DSO symptom clusters were evaluated ≥ 2, and at least one area of life was interfered with DSO symptoms. Previous research confirmed the validity of the ITQ-CA in the sample of Lithuanian adolescents (Kazlauskas et al., 2020). In this study, the internal consistency of ITQ-CA items was acceptable at T1 and good at T2 (Cronbach’s alpha of T1/T2 = .70/.90).

#### Stressors

The Adjustment Disorder New Module Child and Adolescent version – 8 Stressors list (ADNM-CA-8) (Abe et al., 2022) was used to measure exposure to stressors in the past year. The ADNM-CA-8 is an adapted version for children and adolescents of the ADNM-8 adult version (Kazlauskas et al., 2018). The stressors list comprises 16 interpersonal, school- and health-related stressors (e.g., parental divorce, severe disease, bullying). Participants were asked whether they experienced any of these stressors, using dichotomous *yes/no* answers. The total number of experienced stressors was used in this study. The ADNM-CA-8 has been tested previously in Lithuania in a cross-cultural sample (Abe et al., 2022).

#### Somatic symptoms

Children’s Somatic Symptoms Inventory-8 (CSSI-8) is an 8-item measure for somatic distress – the extent to which youth are bothered by various nonspecific somatic symptoms (Walker et al., 2009). The current study was the first time CSSI-8 had been used in the Lithuanian adolescent sample. The CSSI-8 was translated into Lithuanian and then back-translated by independent translators and confirmed by the authors of the CSSI-8.

Participants were asked how much in the past 2 weeks they have experienced somatic symptoms, such as headache, stomach ache, dizziness, and others, with the possible answers on a five-point Likert scale from *Strongly disagree* (= 0) to *Strongly agree* (= 4). The total score of CSSI-8 was calculated by adding the values of all eight items, and may range from 0 to 32. Higher CSSI-8 scores indicate more severe somatic symptomatology. Missing data were handled by making the average of all answered items and multiplying by 8 (Walker et al., 2009). The internal consistency of CSSI-8 items was acceptable at T1 and good at T2 (Cronbach’s alpha of T1/T2 = .72/.83).

#### Social support

Social support was measured using the Perceived Positive Social Support (PPSS) scale and seven additional questions about specific people to whom adolescents can talk when he or she is experiencing difficulties. PPSS was developed by revising Crisis Support Scale (Joseph et al., 1992). The PPSS comprises four items measuring instrumental (if the person has an opportunity to share with another person about experienced difficulties) and emotional (if the person experiences empathy and help from other people regarding their difficulties). Each item was evaluated using a seven-point Likert scale ranging from *Never* (= 1) to *Always* (= 7). The total score is the sum of the responses to all four items, with higher scores indicating receiving more perceived social support. The PPSS was used only at T2 measurement, and the internal reliability of the PPSS was good (Cronbach’s alpha = .86).

Also, participants were asked at T1 and T2 about people with whom participants could talk about their difficulties. The multiple-choice answer was given, with the options: (1) father, (2) mother, (3) other family members, (4) a friend, (5) nurse, (6) teacher or other adults at school, (7) other adults, (8) nobody. Each answer *yes* was coded as “1” and *no* as “0”. The total number of supportive people was the sum of answers from 1 to 7.

#### Loneliness

The three-item Loneliness scale (Hughes et al., 2004) was used to assess experienced loneliness. Scale measures how often participants feel like they are missing being with other people, are left behind others, and are isolated from others. A three-point scale from *Never* (= 0) to *Often* (= 2) was used for each question. A loneliness score was obtained by summing responses to all three items. Higher scale scores indicate higher loneliness. Loneliness was measured only at T2, and the internal reliability of the items was good (Cronbach’s alpha = .82).

#### Bullying

Bullying was evaluated using three items. The first item measures how often the participant has experienced bullying by him/herself, the second item measures if the participant has witnessed bullying, and the third is if the participant was bullying another person. Each item was evaluated on a 5-point Likert scale, from *Never* (= 0) to *Several times a week* (= 4). Each item, as a different type of experience, was analyzed separately. Exposure to bullying was considered if a response to the item was ≥ 1 (1 = *Once or twice*).

### Data analyses

Chronic complex PTSD was identified if the participant met complex PTSD diagnostic criteria at T1 and T2. Recovery was coded if the participant met the criteria for complex PTSD at T1 and did not meet the criteria for PTSD and complex PTSD at T2. The number of participants who met PTSD criteria at T2 was too small (*n* = 6) to apply statistical methods for comparison with other groups; therefore, data analysis focused on comparing recovery versus chronic complex PTSD groups. Descriptive data of participants who met PTSD criteria at T2 are presented in the supplementary materials (Table S1). Due to the relatively small study sample, we could not utilize multivariate statistical methods for our analyses. The normality tests of continuous variables showed acceptable data fit (skewness and kurtosis range +/−2 (Mishra et al., 2019)). Therefore, univariate statistics were applied to identify differences at T1 and T2 within chronic complex PTSD and recovery groups, and between chronic complex PTSD and recovery groups at T1 and T2 measurement points. Chi-square tests to evaluate sociodemographic differences between chronic complex PTSD and recovery groups were used.

## Results

Overall, 36.4% (*n* = 24) of the study sample has been identified as having chronic complex PTSD over 2 years, 10% met the criteria for PTSD at a 2-year follow-up, and 54.5% (*n* = 36) recovered. We found no significant differences in sociodemographic characteristics of the chronic complex PTSD versus recovery groups, including gender, age, parental education, employment status, or financial situation.

Chronic complex PTSD was associated with exposure to potentially traumatic events (PTEs) and life stressors (See Table 2). At baseline (T1), there were no significant differences (*p* = .245) in lifetime traumatic experiences between the chronic complex PTSD versus recovery groups, with 3.96 and 3.61 PTEs in chronic complex PTSD and recovery groups, respectively. However, adolescents with chronic complex PTSD at 2-year follow-up reported experience of significantly (*p* = .008) more traumatic events over the 2 years (3.08), in comparison to the recovery group (1.72) (See Table 2). The most prevalent PTEs at T1 were serious accidents or injury (*n* = 36; 54.5%), witnessing physical abuse in the community (*n* = 31; 47.0%), and scary medical procedures (*n* = 38; 57.6%). The most prevalent traumatic experiences in chronic complex PTSD and recovery groups were the same as in the total sample and did not differ between these groups statistically at the T1 time point.

**Table 2.** Comparison of chronic complex PTSD and recovery groups at baseline (T1) and a 2-year follow-up (T2) (*N* = 60).

Variable	Chronic complex PTSD *n* = 24		Within group	Recovery *n* = 36		Within group	Between groups	
	T1	T2		T1	T2		T1	T2
	*M* (*SD*)	*M* (*SD*)	*t* (23)	*M* (*SD*)	*M* (*SD*)	*t* (35)	*t* (58)	*t* (58)
Trauma exposure	3.96 (1.71)^a^	3.08 (2.60)^b^	-	3.61 (2.02)^a^	1.72 (1.68)^b^	-	−0.69	−2.48^**^
Life-stressors^c^	5.55 (2.04)	5.18 (2.74)	0.50	5.18 (2.33)	3.32 (1.90)	4.41^***^	−0.61	−3.39^**^
Somatic symptoms	19.00 (4.68)	19.20 (6.39)	−0.14	15.08 (6.03)	10.72 (5.78)	5.01^***^	−2.69^**^	−5.23^***^
PTSD symptoms	15.79 (3.03)	16.54 (4.09)	−0.89	14.22 (4.19)	8.17 (4.94)	6.08^***^	−1.58	−6.88^***^
DSO symptoms	18.67 (4.32)	17.46 (3.78)	1.03	15.31 (3.72)	9.28 (4.98)	7.59^***^	−3.22^***^	−6.83^***^
CPTSD symptoms	34.46 (5.78)	34.00 (7.22)	0.25	29.53 (6.20)	17.44 (7.92)	7.66^***^	−3.10^***^	−8.21^***^
Social support network^d^	1.13 (0.97)	1.08 (1.14)	0.13	1.94 (1.49)	1.81 (1.43)	0.60	2.54^*^	2.07^*^
Loneliness	-	4.62 (1.66)	-	-	2.58 (1.77)	-	-	−4.47^***^
Positive social support	-	15.54 (5.93)	-	-	18.83 (6.15)	-	-	2.06^*^

Note. *p ≤ .05; **p ≤ .01; ***p ≤ .001.

^a^= lifetime trauma exposure at T1

^b^= 24 months trauma exposure at T2.

^c^= two cases with missing data

^d^= one case with missing data.

The most prevalent PTEs at T2 timepoint in the chronic complex PTSD group were serious accident or injury (*n* = 10; 41.7%), sudden or violent death of close one (*n* = 9; 37.5%), and stressful or scary medical procedure (*n* = 11; 45.8%). The most common PTEs in the recovery group were serious accidents or injury (*n* = 17; 47.2%), physical violence experienced not from a family member (*n* = 7; 19.4%), and witnessed physical violence in the community (*n* = 10; 27.8%). In the chronic complex PTSD group significantly higher prevalence of experiences of someone older touching private parts (0.0% in recovery vs. 29.2% in chronic complex PTSD, χ^2^ (1) = 11.89, *p* < .001), sudden or violent death of close one (11.1% in recovery vs. 37.5% in chronic complex PTSD, χ^2^ (1) = 5.91, *p* = .015), and stressful or scary medical procedures (11.1% in recovery vs. 45.8% chronic complex PTSD groups, χ^2^ (1) = 9.26, *p* = .002) have been found.

Furthermore, while the chronic complex PTSD group and recovery group at baseline reported similar levels of life-stressors exposure over the last year (*p* = .273), the recovery group reported significantly fewer life stressors at a 2-year follow-up (*p* < .001) (See Table 2). The most prevalent life stressors in both studied groups at T1 and T2 were an experience of the end of the friendship (76.7% at T1 and 61.7% at T2) and difficulties at school (90.0% at T1 and 85.0% at T2). Also, a highly prevalent life stressor in the chronic complex PTSD group was family conflicts (83.3% at T1 and 75.0% at T2).

We found that the chronic complex PTSD group, in comparison to the recovery group at baseline, had higher levels of complex posttraumatic stress symptoms (*p* = .001; between-group Cohen’s *d* = .82) and somatic symptoms (*p* = .005; between-group Cohen’s *d* = 0.71) (See Table 2). However, only DSO symptoms were higher at baseline (*p* = .001) and not PTSD symptoms (*p* = .060) in the chronic complex PTSD group versus the recovery group (See Table 2).

The chronic complex PTSD group experienced more loneliness, had a smaller social support network in terms of how many close ones were supporting them, received less overall positive social support (See Table 2), and experienced more bullying at school, 11% in recovery versus 37.5% in chronic complex PTSD group, χ^2^ (1) = 5.91, *p* = .015. However, groups did not differ regarding witnessing bullying in school or being bullies. The chronic complex PTSD group also received more professional psychological support between T1 and T2 measurements (See Table 1).

## Discussion

This observational study explored changes in complex PTSD diagnostic status in adolescence over 2 years and searched for factors associated with chronic complex PTSD course versus recovery from complex PTSD in a general population-driven sample. All participants met the diagnostic criteria for complex PTSD at baseline, and our study found that 54% of adolescents recovered in 2 years from complex PTSD; however, 36% had a chronic course of complex PTSD, and 10% met the criteria for PTSD at a 2-year follow-up.

Exposure to traumatic events and life stressors over 2 years was associated with chronic complex PTSD. Participants with more recent trauma exposure and also more recent life stressors had a higher risk of long-term complex PTSD symptoms. Furthermore, at the baseline, the recovery and the chronic complex PTSD were similar in lifetime trauma or recent life-stressors exposure. However, the differences between recovery and a prolonged course of complex PTSD emerged in comparing negative life experiences over the last 2 years. Chronic course of complex PTSD was associated with a higher prevalence of various traumatic events, specifically such events as the loss of a loved one, scary medical procedures, or sexual abuse. Overall, these findings are in line with a theoretical conceptualization of PTSD and complex PTSD, and the findings of other studies (Redican et al., 2022). However, more longitudinal studies are needed to estimate the role of negative life experiences on children and adolescents with complex PTSD.

Further, in line with previous studies, we found that social factors in adolescence are particularly important for understanding the development and recovery of complex PTSD (Daniunaite et al., 2021; Kazlauskas et al., 2022). Our longitudinal study demonstrated that a low social network of support, lack of positive social support, loneliness, and bullying experiences contributed to a prolonged course of complex PTSD. We also found that adolescents with chronic complex PTSD symptom trajectory had more somatic symptoms than the recovery group. The recovery and chronic complex PTSD groups were similar in somatic symptoms at baseline. Complex PTSD symptoms declined in the recovery group, along with somatic symptoms over 2 years. However, somatic symptoms were high and stable in the chronic complex PTSD group. It has been previously indicated that somatization could have an important role in the clinical picture of complex PTSD (Van Der Kolk et al., 2005); however, this finding should be further explored in future studies.

Our sample was obtained from a general population of adolescents in public schools. Thus this was not a clinical or treatment-seeking sample. Sadly enough, only a few of traumatized adolescents received psychological treatment for their trauma-related difficulties based on adolescents self-reports. Surprisingly, only around one out of 10 adolescents in the recovery group reported receiving professional support over the studied timeframe. This may indicate that recovery from complex PTSD was not associated with receiving therapy but instead was facilitated by a benevolent social environment that provided social support and prevented exposure to traumatic events. Around half of the chronic complex PTSD sample reported they had received at least some professional support, which implies that caregivers or educators identify, at least to some extent, complex PTSD-related emotional and behavioral difficulties and refer to relevant services.

This observational study was organized so that researchers could not identify the study participants; therefore, the research team was blinded for the disorders in the studied sample. We provided information for all adolescents on the available care options at school and community during the data collection at baseline. The study did not explore what mental health or counseling services adolescents received, which would be important to study in the future. However, the study provided important information about a lack of services for traumatized youth, particularly those with high levels of complex PTSD symptoms. Previous studies indicated low acknowledgment of PTSD and significant barriers to trauma-focused treatment in Lithuania’s health care (Kazlauskas et al., 2017). Our findings raise concern about the need for better mental health services and care for trauma-exposed adolescents.

### Limitations

Despite interesting findings, our study should be viewed in light of limitations that might have had an impact on the results of our study. Only two assessment points with a 2-year gap were included in the study. It is possible there were fluctuations in complex PTSD symptoms over 2 years, which were not evaluated due to the methodology of our study. In collecting retrospective data on trauma exposure at baseline and follow-up, we used a screening tool that did not include a specific time for exposure of each PTEs. At follow-up, we only asked whether the participant had experienced the listed PTEs over the last 2 years. The data on when participants were exposed to specific traumatic events could provide valuable information on the development and trajectories of complex PTSD symptoms, and should be included in future studies. Further, we used self-report measures for a probable complex PTSD diagnosis, and clinical measures using diagnostic interviews could provide more reliable data on complex PTSD diagnostic status. However, to the best of our knowledge, at the time of the study, no diagnostic interviews for complex PTSD were available for adolescents, as complex PTSD is still a new diagnosis in ICD-11. Finally, the sample size was small, and drop-out was relatively high in this study. We managed to reach only about 61% of the target sample who were identified with complex PTSD at the baseline. Participants were collaborative and willing to participate. The reasons for the drop-out in the study were associated with administrative issues rather than an active refusal to participate, which included changing schools, moving to live in other areas, the COVID-19 pandemic restrictions-related difficulties in accessing school facilities.

### Conclusion

The study adds valuable data on the clinical picture of complex PTSD in adolescence. As the current definition of complex PTSD in ICD-11 is relatively new, there is undoubtedly a lack of understanding of the trajectories of symptoms of complex PTSD from a lifespan perspective. Our study informs that prolonged trauma exposure and life stressors contribute to ongoing complex PTSD symptoms that could last at least 2 years. Further, the study shows that the social context of children and adolescents is very important, and social problems, such as bullying at school or lack of positive social support in traumatized youth, prolong complex PTSD symptoms. The findings of the study are relevant to clinical practice and further development of interventions targeting complex PTSD in youth.

## Supplemental Material

Supplemental Material - Complex posttraumatic stress disorder in adolescence: A two-year follow-up study Supplemental Material for Complex posttraumatic stress disorder in adolescence: A two-year follow-up study by Evaldas Kazlauskas, Agniete Kairyte and Paulina Zelviene in Clinical Child Psychology and Psychiatry
