# Supplementary material for: Complex posttraumatic stress disorder in adolescence: A two-year follow-up study
Source: Clin Child Psychol Psychiatry. 2023 Jul 4;29(2):466–78. doi: 10.1177/13591045231187975 (PMC10945978; doi:10.1177/13591045231187975)
Supplement: Supplemental Material - Complex posttraumatic stress disorder in adolescence: A two-year follow-up study [file sj-pdf-1-ccp-10.1177_13591045231187975.pdf]

Supplementary material

Table S1

Descriptives of variables of participants who changed diagnostic status from complex PTSD at baseline (T1) to PTSD at a two-year follow-up (T2) (*n* = 6).

Variable	PTSD group	
	T1	T2
	M (*SD*)	*M* (*SD*)
Trauma exposure	4.83 (3.06)[a]	2.17 (1.60)[b]
Life-stressors[†]	5.40 (2.04)	4.17 (2.64)
Somatic symptoms	18.50 (2.89)	13.50 (4.85)
PTSD symptoms	15.00 (4.05)	14.67 (4.08)
DSO symptoms	17.00 (3.29)	8.67 (2.73)
CPTSD symptoms	32.33 (3.80)	23.33 (4.80)
Social support network	1.17 (0.75)	0.83 (0.75)
Loneliness	-	3.67 (0.52)
Positive social support	-	18.50 (3.27)

Note. [a] = lifetime trauma exposure at T1; [b] = 24 months trauma exposure at T2.
[†] = one case with missing data
